# Bone Marrow-Derived Mesenchymal Stem Cells Drive Lymphangiogenesis

**DOI:** 10.1371/journal.pone.0106976

**Published:** 2014-09-15

**Authors:** Ludovic Maertens, Charlotte Erpicum, Benoit Detry, Silvia Blacher, Bénédicte Lenoir, Oriane Carnet, Christel Péqueux, Didier Cataldo, Julie Lecomte, Jenny Paupert, Agnès Noel

**Affiliations:** 1 Laboratory of Tumor and Development Biology, Groupe Interdisciplinaire de Génoprotéomique Appliquée - Cancer (GIGA-Cancer), University of Liège, Liège, Belgium; 2 Laboratory of Cardiovascular Research, Centre de Recherche Public de la santé (CRP-santé), Luxembourg, Luxembourg; Rutgers - New Jersey Medical School, United States of America

## Abstract

It is now well accepted that multipotent Bone-Marrow Mesenchymal Stem Cells (BM-MSC) contribute to cancer progression through several mechanisms including angiogenesis. However, their involvement during the lymphangiogenic process is poorly described. Using BM-MSC isolated from mice of two different backgrounds, we demonstrate a paracrine lymphangiogenic action of BM-MSC both *in vivo* and *in vitro*. Co-injection of BM-MSC and tumor cells in mice increased the *in vivo* tumor growth and intratumoral lymphatic vessel density. In addition, BM-MSC or their conditioned medium stimulated the recruitment of lymphatic vessels *in vivo* in an ear sponge assay, and *ex vivo* in the lymphatic ring assay (LRA). *In vitro*, MSC conditioned medium also increased the proliferation rate and the migration of both primary lymphatic endothelial cells (LEC) and an immortalized lymphatic endothelial cell line. Mechanistically, these pro-lymphangiogenic effects relied on the secretion of Vascular Endothelial Growth Factor (VEGF)-A by BM-MSC that activates VEGF Receptor (VEGFR)-2 pathway on LEC. Indeed, the trapping of VEGF-A in MSC conditioned medium by soluble VEGF Receptors (sVEGFR)-1, -2 or the inhibition of VEGFR-2 activity by a specific inhibitor (ZM 323881) both decreased LEC proliferation, migration and the phosphorylation of their main downstream target ERK1/2. This study provides direct unprecedented evidence for a paracrine lymphangiogenic action of BM-MSC via the production of VEGF-A which acts on LEC VEGFR-2.

## Introduction

Mesenchymal Stem Cells (MSC) originating from different tissues or organs are multipotent progenitor cells that have the capacity of self-renewal and differentiation into different cell types of the mesenchymal lineage such as chondrocytes, osteoblasts, adipocytes, fibroblasts and endothelial cells [1]–[3]. Although bone marrow (BM)-derived MSC (BM-MSC) reside predominantly in the BM, these cells migrate to distant sites with a tropism for inflamed or injured tissues [4], [5], primary tumors and pre-metastatic niches [6], [7].

Co-injection experiments of MSC and tumor cells already provided evidence that BM-MSC promote tumor growth [8], [9] and drive cancer cell invasion [10], [11]. Although the precise molecular mechanisms are not fully elucidated, different features and properties of these multipotent plastic cells are likely contributing to their tumor promoting effect. MSC have been reported to promote cancer cell proliferation, survival and invasion by releasing, at least, trophic factors, cytokines and remodeling enzymes (matrix metalloproteinases, serine proteases) [10]–[13]. They can differentiate into fibroblast-like cells and thereby contribute to the generation of carcinoma-associated fibroblasts, which emerged as key contributors of the tumor microenvironment permissive for tumor progression and metastatic dissemination [14]–[16]. In a murine skin carcinoma model, we recently demonstrated that alpha-smooth muscle actin positive cells issued from BM-MSC are the unique source of matrix metalloproteinase-13, a stromal mediator of cancer cell invasion [11]. These data support the concept of fibroblast subset specialization depending upon their cellular origin. Accumulating evidences also demonstrate that BM-MSC promote angiogenesis through the recruitment of endothelial progenitor cells [17], the differentiation into endothelial cells and pericyte-like cells [18], [19], the secretion of soluble angiogenic factors such as Vascular Endothelial Growth Factor(VEGF)-A or basic Fibroblast Growth Factor (bFGF), and the release of exosomes as well [20]–[23].

In addition to blood vessels, the lymphatic vascular system plays an essential role in physiological fluid homeostasis, inflammation and cancer metastasis [24]–[26]. VEGF-C is viewed as the more potent lymphangiogenic factors, mainly by activating the VEGF Receptor (VEGFR)-3 a tyrosine kinase receptor expressed on lymphatic endothelial cells (LEC) [27], [28]. VEGFR-2 has been also detected on LEC, supporting the role of VEGF-A/VEGFR-2 axis in lymphangiogenesis [29], [30]. Additional growth factors regulating the lymphangiogenic process include VEGF-D, FGF, platelet-derived growth factor, epidermal growth factor [31], angiopoietins [32] and transforming growth factor beta family members [33], [34]. By analogy with angiogenesis, the contribution of BM-MSC in lymphangiogenesis is anticipated [21], [35], [36], but poorly documented.

This study aimed at investigating the heterotypic interactions between BM-MSC and the lymphatic network. We are providing evidence that BM-MSC stimulate lymphangiogenesis in physiological and pathological (malignant tumor) conditions primarily by secreting VEGF-A and activating VEGFR-2 pathway.

## Materials and Methods

### Cells and reagents

Luciferase-expressing Lewis Lung Carcinoma (LLC-Luc) cell line of C57BL/6 mouse origin was purchased from Caliper Lifesciences. LLC-Luc cells were cultured in DMEM (Gibco, Gent, Belgium) supplemented with 10% heat-inactivated fetal bovine serum (FBS) (Gibco, Gent, Belgium), 2 mM glutamine (Gibco, Gent, Belgium), 100 UI/ml penicilline/streptomycin (Gibco, Gent, Belgium), 1 mg/ml geneticin (Serva, Heidelberg, Germany) and maintained in a humidified incubator at 37°C and in a 5% CO_2_ atmosphere. Two type of LEC were used in this study: HMVEC-dLy (Lonza, Braine-l'Alleud, Belgium) and Human telomerase-transfected dermal LECs (hTERT-HDLECs) [37]. LEC were cultured in EGM2-MV medium (Lonza, Braine-l'Alleud, Belgium) until confluence was reached.

### Mice

Six weeks old female C57BL/6 mice purchased from Janvier (Saint Berthevin, France) and transgenic mice heterozygous for the enhanced green fluorescent protein under the control of ß-actin promoter C57BL/6-Tg(ACTbEGFP)10sb (Jackson Laboratories, Bar Harbor, ME) were used throughout this study. The animals were maintained with a 12-hour light-dark cycle and had free access to food and water. Experimental procedures were approved by the Animal Ethical Committee of the University of Liège (Liège, Belgium) and all animal experiments were performed in compliance with the Animal Ethical Committee of the University of Liège (Liège, Belgium).

### BM-MSC isolation and characterization

Two independent BM-MSC populations were isolated from the bone marrow and compact bones of either C57 BL/6J or C57BL/6-Tg(ACTbEGFP)10sb mice (8–10 week old). The mouse femurs and tibias were crushed with mortar and pestle in phosphate buffer containing 2% FBS and 1 mM EDTA (Merck, Overijse, Belgium). Cell suspension was collected and the remaining bone fragments were incubated at 37°C in 0.25% collagenase 1A (Sigma-Aldrich, St-Louis, MO) in Phosphate Buffer Saline (PBS) containing 20% FBS. After 45 minutes (min) of incubation, cells were harvested, pooled with the initial cell suspension. Mononuclear cells were isolated by using 1,073 mg/ml Ficoll, (GE Healthcare Bioscience, Diegem, Belgium) by centrifugation at 352×g for 45 min at 4°C. Cells were rinsed twice with PBS and then seeded in complete Mesencult medium (Stem Cells, Technologies, Grenoble, France). After 3 days of culture at 37°C under mild hypoxic condition (5% O_2_, 5% CO_2_, 90% N_2_), non adherent cells (hematopoietic cells) were removed and the adherent layer was cultured until it reached 70–80% confluence. Mesenchymal cell population was further purified by negative selection with “*mouse hematopoietic progenitor Stem Cell enrichment set*” (BD Falcon, San Jose, California). Unwanted cells were targeted for removal with biotinylated antibodies directed against markers of non-MSC cells (CD3e, CD11b, CD45, Ly-6G, Ly-6C and ly-76). Those labeled cells were recognized by streptavidin particles and separated by using a magnet (Adem-Mag MSV, Ademtech, Pessac, France), while desired unlabeled cells were collected. Cells were checked for BM-MSC marker expression (CD106^+^, Sca1^+^, CD34^−^, CD45^−^, CD11b^−^) by flow cytometry and for their capacity to differentiate into adipocytes, osteocytes and chondrocytes, as previously described [38]. BM-MSC were used between passages 5 and 10.

### BM-MSC conditioned medium

BM-MSC were seeded and cultured in mouse MesenCult medium (STEMCELL Technologies, Grenoble, France) until 90% confluence under mild hypoxic condition (5% O_2_, 5% CO_2_, 90% N_2_). Then, culture media were replaced by serum-free EBM-2 (Lonza, Braine-l'Alleud, Belgium) and cells were placed under normoxic condition. The supernatant of 24 h incubation was collected, centrifuged at 1,000 g for 10 min and concentred with Amicon Ultra Centrifugal Filters (Millipore, Carrigtwohill, Ireland) and aliquots of the conditioned medium were stored at −80°C until use.

### Tumor transplantation model

Mice were anesthetized by intraperitoneal injection of ketamine hydrochloride (75 mg/kg body weight; CEVA, Bruxelles, Belgium) and xylazine (10 mg/kg body weight; VMD, Arendonk, Belgium) and LLC-Luc cells (5×10^4^ cells) were inoculated alone (n = 30) or mixed with BM-MSC (2,5×10^5^ cells) (n = 25). Cells were injected between the skin and cartilage on the dorsal side of each mice ear in a final volume of 20 µl of serum-free DMEM. After 21 days, mice were checked by *in vivo* bioluminescence imaging before being sacrificed. Twelve min before imaging, luciferin (3 mg/100 µl) (Promega, Madison, WI) was intraperitonealy injected into mice. Mice were anesthetized with isoflurane/oxygen (Abbott, Wavre, Belgium) and ventral images were collected for 10 sec to 1 min using the IVIS imaging system (Caliper Lifesciences, Hopkinton, MO). Photons emitted from the tumor were quantified using LivingImage software (Caliper Lifesciences, Hopkinton, MO). Tumor tissue samples embedded in paraffin were cut at 5 µm thick using a microtome (Leica, Diegem, Belgium). Deparaffinized and rehydrated sections were treated by autoclave to ensure epitope exposition and incubated 20 min, at room temperature with H_2_O_2_ 3% (Merck, Overijse, Belgium) to block endogenous peroxydases. After brief H_2_O washes, slides were blocked with 10% BSA during 1 h at room temperature. Antibodies raised against podoplanin (1/750; R&D Systems, Abingdon, UK) were incubated for 1 h at room temperature. After washes in PBS, sections were incubated for 30 min with rabbit anti-goat/biotin (1/400; E0466, Dako, Glostrup, Denmark) followed by washes and 30 min incubation with Streptavidin/HRP (1/500; P0397, Dako, Glostrup, Denmark). After brief PBS wash, the antibody-antigen complex was visualized by treatment with 3, 3′-diaminobenzidine (DAB, Dako, Glostrup, Denmark) at room temperature and sections were rinsed in H_2_O. Sections were counterstained with Hematoxylin/eosin, dehydrated by successive washes in alcohol 70%, 90%, 100%, xylol an mounted in Q Path Coverquick 3000 (Labonord, Templemars, France). Lymphatic vessels and the contour of the tumor were drawn manually for each section. Then, the total area occupied by vessels as well as the area of the tumor sections was measured automatically. Finally, lymphatic vessel density, defined as the ratio between the area occupied by lymphatic vessels and the area of the tumor section, was determined. Image measurements were conducting using the image analysis toolbox of the Matlab 7.9 software.

### Ear sponge assay

Gelatin sponges (Pfizer, Ixelles, Belgium) were cut in small pieces (3 mm^3^), incubated with serum-free EBM-2 or with MSC conditioned medium 20× concentrated with Amicon Ultra Centrifugal Filters (Millipore, Carrigtwohill, Ireland) and embedded in interstitial type I collagen gel (1.5 mg/ml, Serva, Heidelberg, Germany). Small incisions were made on the upper side of the ear and sponges were inserted for 21 days. For sectioning, ears were embedded into Tissu-Teck (Labonord, Templemars, France). Sections were dried at RT for 5 min and incubated successively 2 min in acetone at −20°C and 5 min in methanol 80% at 4°C. After 3 PBS washes, sections were blocked in 1,5% Gloria milk during 30 min and immunostained with polyclonal goat anti mouse lymphatic vessel endothelial receptor-1 (LYVE-1; 1/200; R&D Systems, Abingdon, UK) and Alexa Fluor 488–coupled rabbit anti goat antibody (1/200; Molecular Probes, Gent, Belgium). At least 30 images per experimental conditions were used for computerized quantification. Micrographs of tissue section were digitized in the RGB space from microscope images. In order to quantify lymphatic vessels (in green), RGB images where decomposed into their red (R), green (G) and bleu (B) components. Binary images were obtained in which vessels were represented by white pixels (intensity equal to 1) and the background by black pixels (intensity equal to 0) [39]. On these binary images, we determined the spatial vessel distribution measured from the border of the sponge as previously described [40]. For this purpose, the sponge border was manually delineated and a grid was automatically constructed with the successive dilations (n.1, 2, 3y) of this boundary. The vessel density was then determined on each interval of the grid. Results are expressed (i) in function of the distance to the sponge boundary and (ii) as the number of vessels at a distance of 0.3 mm from the border.

### LRA

Lymphatic ring cultures were performed as described previously [41], [42]. Thoracic duct dissected from C57BL/6 mice was cut into small fragments. The explants were embedded in interstitial type I collagen gel (1.5 mg/mL, Serva, Heidelberg, Germany) and cultured under hypoxic conditions (5% O_2_, 5% CO_2_ and 90% N_2_), in MCDB 131 medium (Invitrogen, Merelbeke, Belgium) supplemented with 4% Ultroser G (BioSepra, Cergy Saint Christophe, France). In some assays, lymphatic rings were confronted to BM-MSC spheroids embedded in the upper layer of collagen gel. To generate multicellular spheroids, BM-MSC were seeded in DMEM medium containing 0.24% high viscosity methyl cellulose (Sigma Aldrich, Saint Louis, MO) (2×10^3^ cells per well) [43]. After 24 h of culture, 4 spheroids were collected and embedded in collagen gels. To test the impact of MSC conditioned medium on lymphatic outgrowth, the culture medium was replaced by 30% MSC conditioned medium supplemented with 70% fresh MCDB-131 medium. As a control, we used a mixture of serum-free EBM-2 and MCDB 131 media (30% and 70%, respectively). Pictures were taken at the indicated times (5–10 days) and computerized quantifications were performed on binary images as described previously [41], [44]. Briefly, a grid composed of concentric rings was generated by successive increments at fixed intervals of explant boundary. Then, the number of microvessel–grid intersections was counted and plotted *versus* the distance from the ring to determine microvessel distribution around the explant. At least 5 images per experimental condition (in duplicate) were used.

### LEC proliferation assay

LEC were seeded in 96-well plates in EGM 2-MV medium at a density of 4×10^3^cells/well. On day 2, medium was replaced with serum-free medium for 4 hours. Wells were washed with PBS and 100 µl/well of EMB-2 control medium or MSC conditioned medium was added in the presence of BrdU (10 µl/ml; Cell Proliferation ELISA, BrdU, Roche, Mannheim, Germany) and 1% FBS. Cells were fixed and stained after 48 h according to manufacturer's instructions (Cell Proliferation ELISA, BrdU, Roche, Mannheim, Germany). For WST-1 assay (Roche, Mannheim, Germany), the same protocol was used. Control medium and MSC conditioned medium were pre-incubated for 1 h at 37°C with 1 µg/ml soluble VEGF Receptor-1, -2 (sVEGFR-1 or -2) (R&D Systems, Abingdon, UK) or ZM 323881 (Tocris Bioscience, Bristol, UK) at a concentration of 10 nM. After a 2 h incubation with WST-1 reagent, sample absorbance was measured according to manufacturer's instructions. Colorimetric analysis was performed with an ELISA reader (Multiskan FC, Thermoscientific, Waltham, MA).

### LEC migration assay

Boyden chamber assay was used for cell migration analysis. Sterile 8-µm pore size polycarbonate filters (Corning Incorporated, New York City, NY) were coated with 100 µl of 0.2% gelatin (Sigma Aldrich, Saint Louis, MO), incubated overnight at room temperature. Filters were hydrated with distilled water 1 h before adding cells. The lower compartment of a 24-well plate was filled with 300 µl of EGM2-MV 2% FBS and 300 µl of control medium or MSC conditioned medium (EGM2-MV 1% FBS final concentration). LEC (5×10^4^) were seeded in the upper compartment in 300 µl of EGM2-MV 0.5% FBS medium. The plate was incubated at 37°C in a humidified atmosphere of 5% CO_2_ and 95% air for 24 h. In some assays, control medium and MSC conditioned medium were pre-incubated for 1 h at 37°C with 1 µg/ml sVEGFR-1 or -2 (R&D Systems, Abingdon, UK). Cells were fixed with methanol for 30 min at −20°C before coloration with Giemsa's Azure Eosin Methylene Blue solution (Merck, Overijse, Belgium) diluted 1/25 in distilled water. The filters were removed and cells on the upper side of the filter were removed gently with a cotton swab. Cell numbers were counted in at least 6 separate fields under a light microscopy (AH3-RFCA, Olympus, Hamburg, Germany) at a 40× magnification.

### Western blotting

LEC were stimulated as described above. In some assays, MSC conditioned medium was pre-incubated for 1 h at 37°C with 1 µg/ml sVEGFR-1 or -2 to trap VEGF-A. To specifically inhibit VEGFR-2, LEC and MSC-conditioned medium were pre-incubated with ZM 323881 (Tocris Bioscience, Bristol, UK) at a concentration of 10 nM for 1 h at 37°C. Cells were rinsed with ice-cold PBS and lysed with RIPA buffer containing phosphatase and protease inhibitors (Roche, Mannheim, Germany). Samples were dissolved in SDS buffer and migrated on 10% SDS-PAGE gel before being transferred onto a PVDF membrane. After 1 h blocking in 1% casein, phosphorylated and total proteins were detected by 4°C overnight incubation with the appropriate antibodies, followed by 1 h incubation in HRP (Horseradish peroxidase)-coupled secondary antibody (Cell Signaling, San Diego, CA) and ECL revelation in LAS4000 imager (Fujifilm, Tokyo, Japan). The following antibodies were used: rabbit monoclonal phospho-ERK1/2, ERK1/2, phospho-VEGFR-2 and VEGFR-2 (Cell Signaling, San Diego, CA). For VEGF-A and -C detection a rabbit polyclonal anti VEGF-A antibody (A-20; Santa Cruz Biotechnology, Dallas, TX) and a rabbit polyclonal anti VEGF-C antibody (104-PA10; ReliaTech, Wolfenbüttel, Germany) were used.

### Immunoprecipitation

After 10 min of stimulation with recombinant human VEGF-A (10 ng/ml), recombinant human VEGF-C (400 ng/ml) (R&D Systems, Abingdon, UK) or MSC conditioned medium, cells were rinsed with ice-cold PBS and lysed with RIPA (Radio-Immunoprecipitation Assay) buffer containing phosphatase and protease inhibitors (Roche, Mannheim, Germany). VEGFR-3 phosphorylated proteins were isolated by binding to antibody directed against phosphotyrosine (1/100; mouse monoclonal anti-phosphotyrosine, Becton Dikinson, Franklin Lakes, NJ), overnight at 4°C. After 4 h precipitation using protein A sepharose beads (GE Healthcare, Diegem, Belgium), proteins were released from the beads by heating 5 min at 95°C in sample buffer (50 mM TrisHCl pH6.8, 4% SDS, 1% beta-mercaptoethanol, 20% glycerol, 0,05% bromophenol blue) and were subjected to VEGFR-2 and -3 Western-Blotting experiments (1/1000; rabbit monoclonal anti-VEGFR-2, Cell Signaling, San Diego, CA and 1/1000; mouse monoclonal anti-VEGFR-3, Millipore, Carrigtwohill, Ireland).

### Statistical analysis

We assessed statistical differences between different experimental groups using Mann-Whitney test, one way ANOVA test or Wilcoxon test for LRA. A p-value <0.05 was considered as significant. Statistical analyses were carried out using the Prism 5.0 software (GraphPad, San Diego, CA).

## Results

### BM-MSC promote lymphangiogenesis in vivo

**Figure 1:**
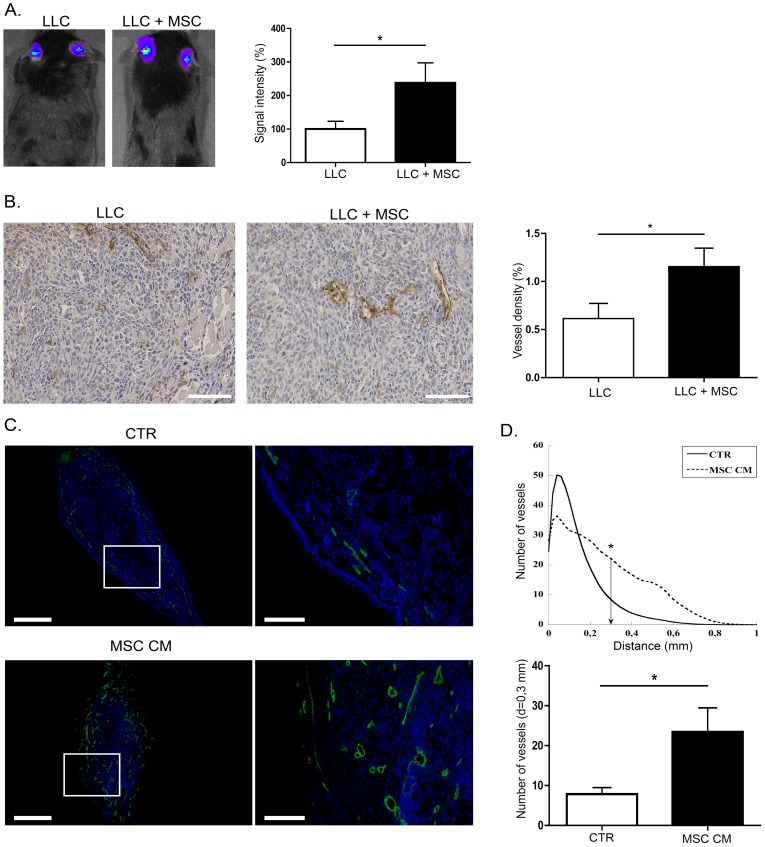
BM-MSC enhance tumor growth and stimulate lymphangiogenesis *in vivo*. (A) *In vivo* bioluminescent signal of tumors developed following injection of 5×10^4^ LLC-Luc cells alone (n = 30) or mixed with 2,5×10^5^ MSC (LLC+MSC) at day 21 (n = 21). The graph corresponds to the quantification of luciferase activity revealing a strong increase of the signal in the LLC+MSC group. (B) BM-MSC enhance lymphatic vessel density in tumors. Sections of tumors induced by injection of LLC-Luc alone (LLC) or with MSC (LLC+MSC) were immunostained with an anti-podoplanin antibody. A computer-assisted quantification of lymphatic vessel density in LLC-Luc tumors (LLC) or in LLC-Luc tumors mixed with BM-MSC (LLC+MSC) is provided on the right. Bar: 100 µm (C) BM-MSC enhance *in vivo* lymphatic vessel recruitment in sponge implanted in mice ear. Sponges soaked with control medium (CTR; n = 8) or with MSC conditioned medium (MSC CM; n = 7) were implanted in mice ear between skin and cartilage. Lymphatic vessels were identified by LYVE-1 immunolabeling (green) and nuclei were evidenced with Dapi (blue). Bars: 5 mm and 1,5 mm on magnification. * P<0.05. (D). The graphs correspond to LYVE-1 positive lymphatic vessel quantification expressed as (1) the number of vessels plotted as a function of distance to the sponge edge (top graph), and (2) the number of vessels at a distance of 0.3 mm from the edge of the sponge (bottom graph). * P<0.05.

Murine BM-MSC isolated from C57Bl/6J or C57BL/6-Tg(ACTbEGFP)10sb mice were characterized by flow cytometry and differentiation assays to assess their MSC phenotype as previously described [38]. Their pro-tumorigenic effects were evaluated *in vivo* by co-injecting luciferase expressing LLC tumor cells with MSC (1∶5 ratio) in mice ears. At day 21, primary tumor growth was monitored *in vivo* by LLC cells-associated luminescence signal quantification using an imaging system IVIS 200 (Figure 1A). Interestingly, the tumor growth was strongly enhanced when tumor cells were mixed with BM-MSC (Figure 1A). As assessed by podoplanin immunodetection on tumor sections and its quantification through a computerized method (Figure 1B), the intratumoral lymphatic vessel density was increased in the presence of BM-MSC (Figure 1B). Next, to determine whether soluble factors secreted by BM-MSC could similarly promote *in vivo* lymphangiogenesis, we implanted a fragment of gelatin sponge soaked with MSC conditioned medium (MSC CM) or control medium (CTR) in mice ear. After 21 days, the development of lymphatic vessels in sponges was releaved by LYVE-1 immunolabeling (Figure 1C). Computer-assisted quantification clearly showed that, when MSC conditioned medium was added, lymphatic vessels infiltrated deeper into the sponge and the number of vessels was significantly higher at a distance of 0.3 mm from the edge of the sponge (Figure 1D).

### BM-MSC promote lymphangiogenesis ex vivo and in vitro

**Figure 2:**
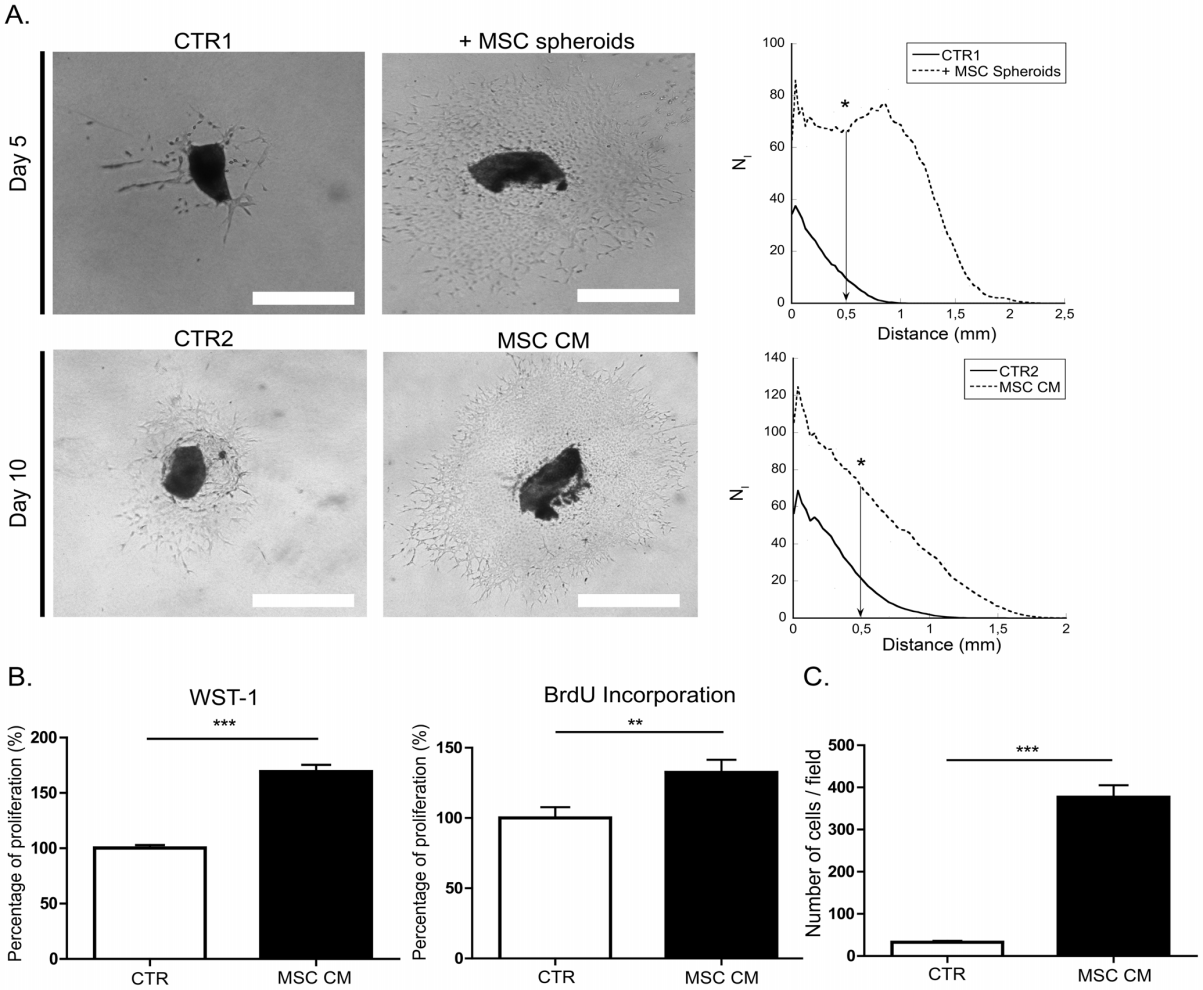
BM-MSC stimulate lymphangiogenesis *in vitro*. (A) Lymphatic rings were cultured during 5 days alone (CTR) or in presence of BM-MSC spheroids (+MSC spheroids), and during 10 days with control medium (CTR2) or with MSC conditioned medium (MSC CM) prepared as described in material and methods section. For quantification, a grid corresponding to successive increments at fixed intervals of explant boundary was used on binarized images and the number of microvessel–grid intersections (N_i_) was quantified on binarized images. Quantification was performed at a distance of 0.5 mm and results are expressed as the number of intersections (N_i_) plotted as a function of distance (mm) to the lymphatic ring. Bar: 500 µm. * P<0.05. (B, C) MSC conditioned medium significantly stimulates the proliferation and migration of LEC *in vitro* as compared to control medium. (B) Proliferation rate was measured by a WST-1 and BrdU incorporation assays. ** P<0.01, *** P<0.001. (C) Migration was measured in a Boyden chamber assay. *** P<0.001.

The impact of BM-MSC on lymphangiogenesis was then evaluated in several *ex vivo* and *in vitro* models reproducing different biological processes associated with lymphangiogenesis [45]. In the lymphatic ring assay, the lymphangiogenesis response was strongly increased when BM-MSC spheroids were embedded in the collagen gel (Figure 2A). Similarly, the addition of MSC conditioned medium resulted in increased lymphangiogenesis compared to control condition (Figure 2A). In 2D LEC cultures, MSC conditioned medium stimulated the proliferation rate of primary LEC (HMVEC-dly cells), as assessed in WST-1 and BrdU incorporation assays (Figure 2B). Finally, MSC conditioned medium enhanced also LEC migration in the Boyden chamber migration assay (Figure 2C). Similar results were obtained with an immortalized lymphatic endothelial cell line (data not shown).

### BM-MSC affect lymphangiogenesis through the release of pro-lymphangiogenic factors

**Figure 3:**
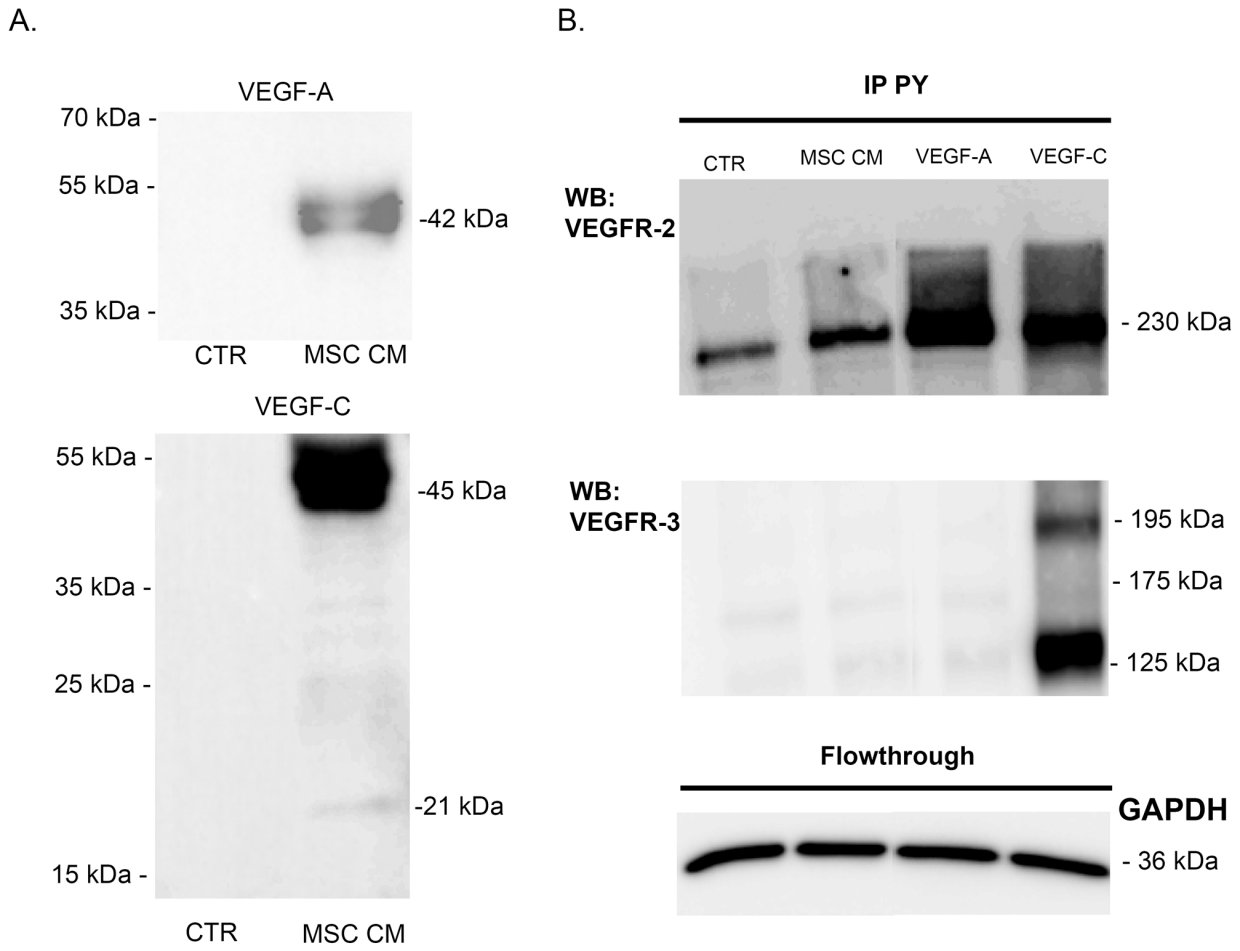
VEGF-A secreted by BM-MSC activate LEC. (A) Western blot analyses of VEGF-A and VEGF-C production on serum-free EBM-2 (CTR) and MSC conditioned medium (MSC CM). (B) VEGFR-2 (top) and VEGFR-3 (bottom) proteins were detected following a phosphorylated tyrosine-containing protein (pY) immunoprecipation (IP) of LEC lysates after cell stimulation with control medium (CTR) or with MSC conditioned medium (MSC CM). Cells treated with VEGF-A (10 ng/ml) or VEGF-C (400 ng/ml) were used as negative and positive controls, respectively. GAPDH western blot was performed on the flowthrough of each sample.

**Figure 4:**
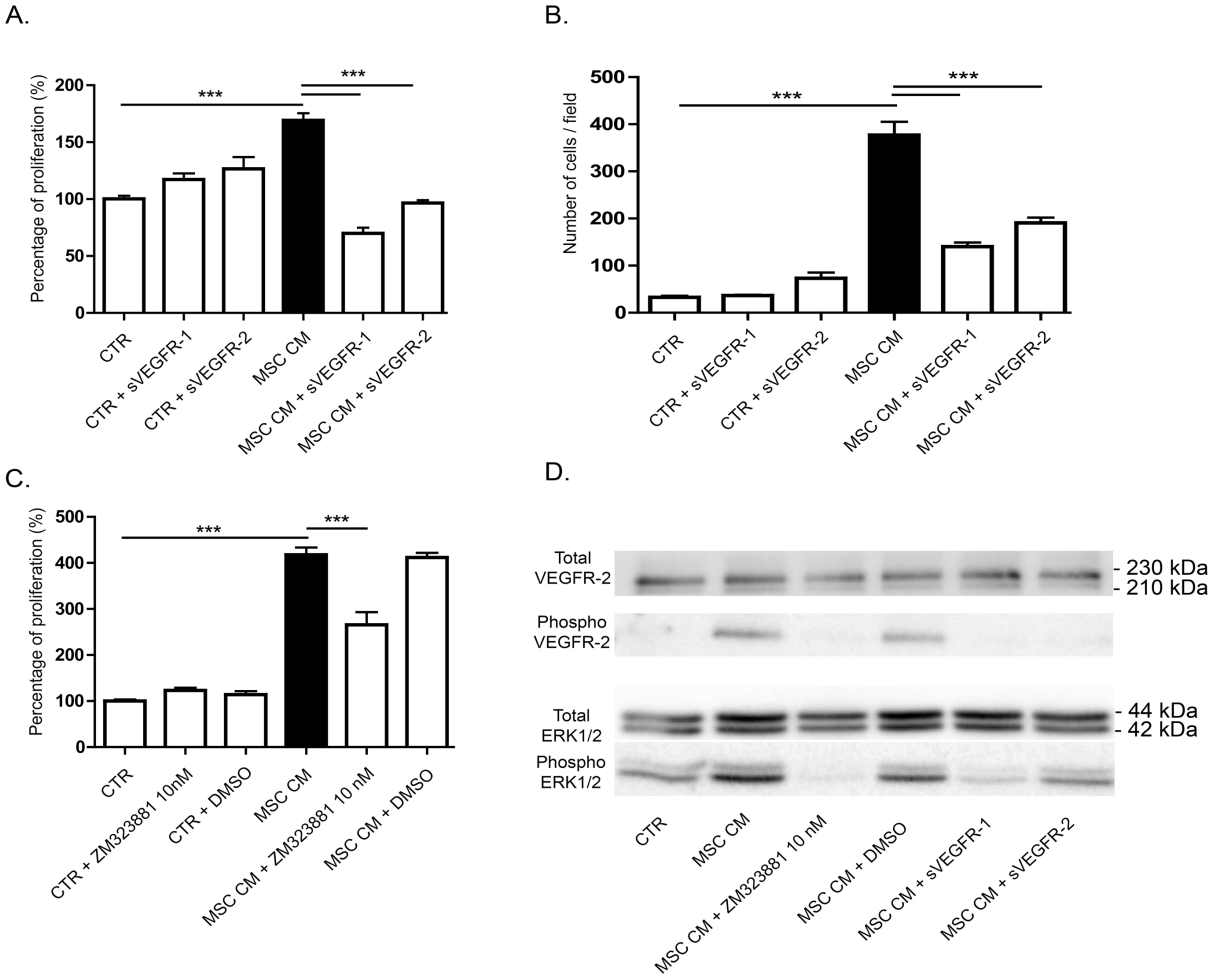
VEGF-A is an important factor implicated in LEC stimulation by MSC conditioned medium. (A, B) The trapping of VEGF-A by the addition of soluble receptors-1 and -2 decreased MSC conditioned medium-induced LEC proliferation, measured by WST-1 assay (A) and migration in a Boyden chamber assay (B). *** P<0.001. (C) Specific inhibition of VEGFR-2 with ZM 323881 10 nM decreased MSC conditioned medium-induce LEC proliferation measured by WST-1 assay. *** P<0.001. (D) Phosphorylation of VEGFR-2 and ERK1/2 analyzed by western blotting on LEC treated or not with soluble VEGF receptors or ZM 323881 10 nM.

VEGF-A and VEGF-C, recognized as the main pro-lymphangiogenic factors are produced by BM-MSC as revealed by western blot performed on their conditioned medium (Figure 3A). Active VEGF-A was detected as a dimer, whereas VEGF-C was mainly secreted as a pro-form with only a very small amount of active VEGF-C. To study the phosphorylation level of their receptors (VEGFR-2 and -3), immunoprecipitation of phosphorylated tyrosine-containing proteins were conducted and VEGFR-2 or VEGFR-3 was detected by western blot. VEGFR-2 phosphorylation was detected upon LEC stimulation with VEGF-A or with MSC conditioned medium (Figure 3B). In sharp contrast, the medium conditioned by MSC did not induce VEGFR-3 phosphorylation. This result is in line with the secretion of VEGF-C mainly in its pro-form, which is unable to activate VEGFR-3 phosphorylation and downstream signaling pathway. These data exclude a key contribution of VEGF-C/VEGFR-3 pathway in our *in vitro* models. Different approaches were used to inhibit VEGF-A in order to assess its functional implication in the BM-MSC-mediated stimulatory effects. The addition of soluble sVEGFR-1 or -2 significantly decreased LEC proliferation rate as well as LEC migration (Figure 4A, B). Incubation of MSC conditioned medium with ZM 323881, which specifically inhibits the transphosphorylation of VEGFR-2, also significantly decreased the LEC proliferation rate (Figure 4C). These data support the concept that VEGF-A/VEGFR-2 pathway is involved in the LEC response to BM-MSC. Notably, MSC conditioned medium triggered the transphosphorylation of VEGFR-2 and increased the phosphorylation of ERK1/2 on LEC (Figure 4D). Incubation of MSC conditioned medium with both sVEGFR-1 and -2 decreased VEGFR-2 and ERK1/2 phosphorylation. In the presence of ZM 323881, the phosphorylation of ERK1/2 was almost inhibited, confirming that VEGF-A/VEGFR-2 axis is the main activator of ERK1/2 pathway on the LEC response observed upon BM-MSC stimulation. All together, these data demonstrate that the pro-lymphangiogenic effect induced by BM-MSC rely on the activation of VEGFR-2 through the VEGF-A secreted by these cells.

## Discussion

Although it is now well accepted that multipotent BM-MSC contribute to cancer progression through different mechanisms, their involvement during lymphangiogenesis is poorly described. In a murine model of LLC-Luc cells injection, we herein demonstrate that the co-inoculation of BM-MSC with LLC-Luc cells increased tumor growth and intratumoral lymphatic vessel density. The MSC-mediated pro-lymphangiogenic effects are further supported by the increased lymphatic vessel formation observed *in vivo* (ear sponge assay) and *ex vivo* (LRA), and by the enhanced proliferation and migration rates of LEC *in vitro* induced by BM-MSC or their conditioned medium. The mechanism was mediated at least through the secretion of VEGF-A by BM-MSC acting on VEGFR-2 expressed by LEC.

The capacity of medium conditioned by BM-MSC to reproduce the effect observed with cells suggested that BM-MSC secreted pro-lymphangiogenic factors, which could act directly on LEC. Surprisingly, despite the presence of the most potent lymphangiogenic factors VEGF-C and -D in the MSC conditioned medium, they do not seem to be implicated in the pro-lymphangiogenic effect observed [22] However, we provide evidence that BM-MSC stimulate lymphangiogenesis through a direct impact on LEC via the secretion of VEGF-A. This concept is supported by the inhibition of LEC proliferation and migration by VEGF-A trapping with sVEGFR-1 or -2. In addition, the key contribution of VEGFR-2 is demonstrated by the use of a specific inhibitor (ZM 323881) of VEGFR-2 phosphorylation, which blocked MSC conditioned medium mediated phosphorylation of VEGFR-2 and of its downstream target ERK1/2. These findings are in line with previous reports showing that VEGF-A stimulates LEC proliferation and migration through the activation of VEGFR-2 [29], [46]. We here demonstrate the functional implication of VEGF-A/VEGFR-2 pathway during BM-MSC mediated lymphangiogenic response. However we cannot exclude a synergistic effect of VEGF-C produced by BM-MSC *in vivo*. Indeed, as assessed by Western blotting, BM-MSC produce mainly pro-VEGF-C, which could be processed into mature VEGF-C *in vivo*. Interestingly, a recent report has identified A disintegrin and metalloprotease with thrombospondin motifs-3 (ADAM-TS3) as a key protease involved in pro-VEGF-C processing [47]. Despite the production of ADAM-TS3 by BM-MSC as assessed by RT-PCR (data not shown), VEGF-C is found in its pro-form. This might be ascribed to the localization of ADAM-TS3 at the cell surface while pro-VEGF-C is secreted in the medium. The observed low amount of active VEGF-C in the medium is in line with the findings of Jeltsch et al [47] on different cell lines.

A pro-angiogenic phenotype of MSC is now well accepted and relies on a putative combination of direct and indirect effects on blood endothelial cells. In addition to acquire a lymphatic phenotype *in vitro* and induce lymphatic regeneration *in vivo* [48], MSC can directly take part to vessel formation by transdifferentiation into endothelial cells and incorporation into the vessel wall [4], [49]. Furthermore, they can secrete several factors implicated in angiogenesis such as VEGF-A, angiopoietin-1 and bFGF [20], [50]. The indirect pro-angiogenic effects are for instance related to the secretion of interleukin-6 by MSC that induces endothelin-1 production by cancer cells and thereby enhances endothelial cell recruitment and activation [51]. We provide herein evidence for a pro-lymphangiogenic effect of MSC through the secretion of soluble factors among which VEGF-A plays a key role. However, we cannot exclude the contribution of inflammatory cells *in vivo*. Indeed, in mice, the inoculation of tumor cells or of the gelatin sponge induces an inflammatory response, which could contribute to or reinforce the lymphangiogenic effect of MSC. In line with this concept, we previously reported, in a corneal lymphangiogenic model, that infiltrating macrophages are key actors of lymphangiogenesis by secreting VEGF-A [46]. Interestingly, Cursiefen et al [52] have shown that VEGF-A-activated macrophages release VEGF-C/-D that contributes to lymphangiogenesis. Therefore, it is conceivable that MSC and macrophages exert synergistic effects on *in vivo* lymphangiogenesis by representing important cellular sources of VEGF-A, which likely participates to a cascade of cell activation. These data underline the implication of a complex network of inflammatory cells and fibroblastic-like cells such as MSC during lymphangiogenic process most often associated with inflammation in pathological conditions such as for instances ocular disease [46] and in cancer [11], [53].

In addition to BM, MSC can originate from other tissues. Recent data indicate that adipose-derived stem cells (Ad-MSC) could also contribute to angiogenesis [22] and lymphangiogenesis [35] via several secreted factors whose individual contribution is not yet well established. Hypoxia appears as a powerful stimulus of VEGF-A production and angiogenic activity of Ad-MSC [54]. Surprisingly, hypoxic Ad-MSC did not regulate lymphangiogenesis in a subcutaneous sponge assay [54]. The present study reporting the production of basal active levels of VEGF-A by BM-MSC under normoxia is consistent with previous works [21], [55]. It extends the multiple functions of BM-MSC to the regulation of lymphangiogenesis. These findings suggest that MSC originating from BM or adipose tissue likely display different properties [1].

## Conclusions

In conclusion, this study provides direct unprecedented evidence for a paracrine lymphangiogenic action of BM-MSC and identifies these cells as an important source of pro-lymphangiogenic VEGF-A which acts on LEC VEGFR-2.
